# Supplementary material for: Estimating the Burden of Stroke: Two-Year Societal Costs and Generic Health-Related Quality of Life of the Restore4Stroke Cohort
Source: Int J Environ Res Public Health. 2022 Sep 5;19(17):11110. doi: 10.3390/ijerph191711110 (PMC9517815; doi:10.3390/ijerph191711110)
Supplement: Supplementary file 1 [file ijerph-19-11110-s001.zip › ijerph-1862957-supplementary S1.pdf]

International Journal of
Environmental Research and Public Health

MDPI

Supplementary S1. Example Cost questionnaire (2 months)

1. Considering the past 2 months, how often did you visit a general practitioner or practice nurse (by phone, at home, or at the general practice)?
--- times (if you did not have any contact, please fill in '0')

2. Considering the past 2 months, how often did you visit a medical specialist (e.g. neurologist, rehabilitation specialist, cardiologist, intensivist)?
--- times (if you did not have any contact, please fill in '0')

3. Considering the past 2 months, how often did you had (rehabilitation) day treatments (for example physiotherapy or a meeting with the psychologist in the hospital or rehabilitation center)?
--- times (if you did not have any contact, please fill in '0')

4. Considering the past 2 months, how often did you visit a paramedic (e.g. physiotherapist, speech therapist, and occupational therapist) or alternative healer (e.g. homeopaths, acupuncturists)?
--- times (if you did not have any contact, please fill in '0')

5. Considering the past 2 months, how often did you visit a mental health worker of a mental healthcare institution (e.g. psychologist / psychotherapist, a social psychiatric nurse and a psychiatrist)?
--- times (if you did not have any contact, please fill in '0')

6. Considering the past 2 months, what prescribed and over-the-counter medications have you taken? If possible, check the packaging of the medicines. You can enter the details (e.g. name and dose) in the overview below.
Name…….. Dose (e.g. 1 times a day 20 mg)………………….
Name…….. Dose (e.g. 1 times a day 20 mg)………………….

7. Considering the past 2 months, how many nights have you spent in a hospital, rehabilitation clinic, nursing home, psychiatric hospital? Number of nights:

- Hospital …..
- Rehabilitation clinic…..
- Nursing home……
- Psychiatric hospital……

(if you did not have any contact, please fill in '0')

8. Considering the past 2 months, how many hours per week did you receive formal home care?
On average --- hours per week (if you did not have any contact, please fill in '0')

9. Considering the past 2 months, how many hours per week did you receive, because of your health condition, help from family or friends?
On average --- hours per week (if you did not have any contact, please fill in '0')

10. Considering the past 2 months, how many days were you unable to perform daily activities, because of your health condition? (e.g. are absenteeism from school, or the inability to do the household)
--- days (if you did not were unable to perform any daily activities, please fill in '0')

11. Considering the past 2 months, how many days were you unable to perform paid labor, because of your health condition?
--- days (if you did not were unable to perform paid labor, please fill in '0')
