# Supplementary material for: Estimating the Burden of Stroke: Two-Year Societal Costs and Generic Health-Related Quality of Life of the Restore4Stroke Cohort
Source: Int J Environ Res Public Health. 2022 Sep 5;19(17):11110. doi: 10.3390/ijerph191711110 (PMC9517815; doi:10.3390/ijerph191711110)
Supplement: Supplementary file 1 [file ijerph-19-11110-s001.zip › ijerph-1862957-supplementary S2.pdf]

International Journal of
Environmental Research and Public Health

MDPI

Supplementary S2. Table Cost prices by categories.

Cost category	Volume	Cost (€)
**Healthcare costs**		
General practitioner	Contact	34.34
Specialist	Contact	117.59
Allied health professionals	Contact	33.82
Mental healthcare professionals	Contact	101.98
Rehabilitation treatments	Day	287.22
Hospital	Overnight stay	668.09
Rehabilitation clinic	Overnight stay	478.70
Nursing home	Overnight stay	174.83
Psychiatric clinic	Overnight stay	314.65
Medication	Various	
**Non-healthcare costs**		
Paid help	Hours	20.81
Informal unpaid care	Hours	14.57
Unpaid labor	Day	116.55
Paid labor		
Men	Day	315.52
Women	Day	263.07

Prices are derived from the Dutch Manual for Cost analyses [1] and indexed to the reference year 2018. Medication were calculated making use of medicijnkosten.nl [2] and daily consumed dosages.

References

1. Zorginstituut Nederland, Guideline for Cost Research: Methodology and Reference Prices for Economic Evaluations in Healthcare (in Dutch), **2016.** Available online: https://www.genesyze.com/resource_guide/zorginstitut-nederland-2016-guideline-economic-evaluations-healthcare-netherlands/ (accessed on 9 September 2019).
2. Medicijnkosten.nl. Available online: https://medicijnkosten.nl/ (accessed on 10 September 2019).
